# Emotional and Behavioral Problems in Children and Adolescents in Central Kenya

**DOI:** 10.1007/s10578-018-0783-y

**Published:** 2018-01-31

**Authors:** Dorcas N. Magai, Jamil A. Malik, Hans M. Koot

**Affiliations:** 1 KEMRI-Wellcome Trust Collaborative Research Programme, Kilifi, Kenya; 2 National Institute of Psychology, Quaid-i-Azam University, Islamabad, Pakistan; 3 Department of Clinical, Neuro- and Developmental Psychology, Amsterdam Public Health Research Institute, Vrije Universiteit Amsterdam, Van der Boechorststraat 1, 1081 BT Amsterdam, The Netherlands

**Keywords:** Emotional/behavioral problems, Child, Adolescent, Kenya

## Abstract

Emotional and behavioral problems (EBP) during childhood and adolescence are a common concern for parents and mental health stakeholders. However, little has been documented about their prevalence in Kenyan children and adolescents. This study aimed to close this gap. The study included Child Behavior Checklist reports from 1022 Kenyan parents on their children (ages 6–18 years) and Youth Self-Reports from 533 adolescents (ages 12–18) living in Kenya’s Central Province. EBP in Kenya are highly prevalent compared to multi-cultural standards for parent reports, with 27 and 17% scoring in the borderline and clinical range, respectively. Based on parent reports, younger children scored higher on EBP than older children, and higher on internalizing problems. Based on self-reports girls scored higher than boys, particularly on internalizing problems. The study provides evidence on elevated parent-reported EBP in Kenyan youths. Mental health providers should focus on interventions that reduce EBP in Kenyan youths.

## Introduction

Emotional and behavioral problems (EBP) of children and adolescents are a common concern for parents and mental health stakeholders alike [1]. Most EBP begin during childhood or adolescence and have consequences for daily life, such as school attendance, ability to learn, substance use, violence and social relations [2], and tend to persist into adulthood [3–5]. The types of problems may differ by the age of the child, and may include a wide variety of problems such as conduct problems, anti-social behavior, anxiety, depression, and substance use, which are among the most prevalent child and adolescent mental health problems. For example, a mismatch between an individual’s desires and societal demands may express itself as difficult behavior in toddlerhood, oppositional behavior in pre-school and school age, aggravate into conduct disorder during late school age and adolescence, and result in crime, delinquency, and substance use in adulthood if not treated as early as possible. Based on a recent meta-analysis, Polanczyk et al. [6] reported a worldwide prevalence of 5.7, 2.6, and 6.5% for any disruptive disorders, any depressive disorders, and anxiety disorders, respectively, in children and adolescents. Thus, worldwide, millions of children and adolescents are affected by mental health problems. However, despite increasing efforts to achieve at a global view on child and adolescent mental health, very little is known on mental health of youth living on the African continent. The dearth of child and adolescent mental health research seems to impede appropriate policy development [7]. This study tries to contribute to closure of this knowledge gap by focusing on EBP and their distribution across age and gender in children and adolescents living in Kenya, one of the continent’s sub-Saharan nations.

Researchers agree that child and adolescent emotional and behavioral problems predict numerous problematic outcomes in adulthood that involve serious challenges across many important life domains including family functioning, mental health, education, and employment [8]. For example, it is well established that oppositional defiant symptoms in childhood may result not only in conduct disorder and antisocial personality disorder, but also predict substance use, risky sexual behaviors, and serious criminal acts in adolescence and adulthood. Vitaro et al. [9] reported that some disruptive children may commit delinquent acts, which makes them two to three times more likely to become violent, serious and chronic offenders, and have longer delinquency careers. Similarly, studies suggest that emotional problems increase with age and place affected children at risk of not only developing anxiety disorders and depressive disorder, but also incur deficiencies in social problem-solving skills, learning problems, academic underachievement, and cognitive deficiencies [10, 11]. Therefore, children who suffer from emotional and behavioral problems are at increased risk of developing future problems that interfere with diverse aspects of their lives which affect their mental health.

Kenya is one of the 48 sub-Saharan African countries that have experienced considerable social tensions and violence [12]. The country covers a surface of about 580,000 square kilometers with a total population of approximately 48 million. The number of children and youths between age 0 and 19 years are estimated to be about 22.5 million [13]. In 2014, the rate of children dropping out of primary schools had been estimated to be 21.5%, which is about 2,139,400 children. In 1975, there were approximately 115 street children in Kenya, however in 2001, this number increased to 250,000. The number of street children has been estimated to be increasing every year [14]. Unemployment rates in youths between 15 and 24 years have been estimated at 24.2%. Additionally, it has been established that youths commit the largest proportion of crime in Kenya. More than half (68%) of the total prison population are youths between 15 and 24 years [15]. In addition, there are recent reports of riots and burning of schools in Kenya [16]. Several factors including poverty, early pregnancies, insecure neighborhoods, poor organization of learning institutions, and adverse life events have been associated with these problems [17].

However, little has been documented about the potential role played by emotional and behavioral problems in the explanation of the high school drop-out rates, criminal behavior, and wandering problems of Kenyan children and adolescents. Even more basically, hardly any robust data are available on overall prevalence of emotional and behavioral problems in Kenyan youths. Harder et al. [18] examined emotional and behavioral problems in impoverished Kenyan youths using the Youth Self-Report (YSR). Their analysis revealed that the proportion of youth classified in the borderline/clinical categories of psychopathology ranged from 1% for rule breaking behavior to 14% for social problems. However, this study may have methodological limitations as the YSR was taken orally in the classroom with all participants present, rather than read and completed individually, which may have influenced the participants’ responses. Secondly, only youth reports were obtained, and reports of other informants were not available. Additionally, this study only focused on impoverished youths, while information from the general population is needed to assess the prevalence of emotional and behavioral problems. A study by Kariuki et al. [19] examined behavior problems in children with epilepsy in rural Kenya using the Child Behavior Questionnaire for Parents (CBQFP). In this study, behavioral problems in 49% of children with epilepsy and 26% of the control group were found. Another study by Weisz et al. [20] compared parent reports of behavioral and emotional problems among children in Kenya, Thailand, and the US using the Child Behavioral Checklist (CBCL). Weisz et al. reported that Kenyan youths rated much higher than youths from the other cultures on emotional and behavioral problems. However, this was only based on the parent reports and no additional informants were involved. Additionally, the study had a small sample of only 90 Kenyan children, limiting the generalizability of its findings. Although these studies give a general view of mental health problems in Kenya, none of them provides precise prevalence rates of emotional and behavioral problems in children and adolescents in Kenya.

Given the limitations of previous studies on EBP in Kenya mentioned above and existing research gaps, the aim of this study was to determine the prevalence of emotional and behavioral problems in Kenyan children and adolescents. The study used parent reports as well as youth self-reports, of which the applicability in this population was tested. It describes the levels of EBP across age and gender in a randomized sample from the general population of children and adolescents in Central Kenya, and compared the prevalence of high levels of EBP to multicultural norms. The study used the CBCL and YSR as parent and adolescent reports, respectively, to assess general psychopathology in this sample. Both instruments are part of an assessment system that originated in the 1960s and are fit to reliably tap general psychopathology from multiple informants as demonstrated in thousands of studies worldwide. Confirmatory factor analyses have supported their syndrome structures in dozens of societies, and multicultural norms are derived from population samples in 57 societies across the world [21].

## Methods

### Samples

The study included Kenyan parents and adolescents living in the country’s Central Province. The data collection was conducted in two counties north of Nairobi, covering both urbanized and rural areas: Kiambu County (population 1.6 million), situated immediately north of the capital, and Nyeri County (population 700,000), located in the Central Highlands of Kenya. Kiambu County has 12 sub-counties, 95 locations, and 236 sub-locations, while Nyeri County has six sub-counties, 73 locations, and 249 sub-locations. Within these counties, stratified random sampling was set up. Using the formula below [22], the number of sub-locations to be sampled in both Kiambu and Nyeri counties were calculated:

$$n=\frac{{N{C^2}}}{{{C^2}+\left( {N - 1} \right){e^2}}}$$

where, n is the required number of locations, N is the total number of locations, C is the coefficient of variation (0.2), e is the margin of error (0.05).

In total 14 sub-locations in Kiambu and 14 sub-locations in Nyeri were selected to get a representative sample in both counties. The 28 sub-locations were randomly sampled from the counties’ sub-locations. In each sub-location stratified sampling based on an equal distribution of target children across age (6–18 years) and gender was used to select participants. A systematic sampling was used to sample every 3rd household that we visited. However, in some areas we used a convenience sampling method to obtain the participants as these places were sparsely populated or it was difficult to obtain the required sample using the systematic random sampling method. Based on power calculations (power = .80, α = .05), the target sample included 1068 parents and 576 adolescents, equally distributed across gender and the 13 age groups (ages 6 through 18 for parent information; ages 12 through 18 for adolescents).

### Measures

#### Demographics

The following demographic variables were assessed for parent and adolescent informants: age, gender, ethnicity, level of education, religious affiliation, civil status (parents only), number of children in the home, parents’ relationship with the child, whether the child visited a school or not, and socio-economic activities.

#### Child Behavior Checklist/4–18 (CBCL)

The CBCL [23] was used to obtain standardized parental reports of children’s problem behaviors. The problem section consists of 118 3-point items describing behavioral and emotional problems. Parents were asked to rate the occurrence of their children’s problem behaviors in the past 6 months by circling 0 (for not true), 1 (for somewhat or sometimes true) or 2 (for very true or often true). For each age- and gender group, the current study used existing factors to represent syndromes of problems that tend to occur together as identified in other studies [23]. These syndromes included Anxious/Depressed, Withdrawn/Depressed, Somatic Complaints, Social Problems, Thought Problems, Attention Problems, Rule-Breaking Behavior, Aggressive Behavior, and the broad-band scales Internalizing Problems, Externalizing Problems, and Total Problems. The degree to which a child manifests problems as reported by the parents is expressed by a score on each scale representing a syndrome scale that is derived empirically [24]. Scale scores are computed by summing all 0, 1 and 2 s for items in the scale. The Swahili version of the CBCL was obtained from the author of the ASEBA and used in this study. The CBCL has worldwide established good psychometric properties (see http://www.aseba.org), and its preschool version has been validated for use in the Kenyan population [25]. Psychometric findings for the sample in this study are reported below.

#### Youth Self-Report (YSR)

The YSR [26] was used to obtain standardized youth self-reports. Its problem section consists of 103 items that describe a broad range of problem behaviors and it was modeled after the CBCL. The YSR has the same format as the CBCL, except that items are worded in the first person. When filling in the questionnaire, the adolescent was asked to score each item that described them at the time of responding to the questions and within the past 6 months. The adolescent was requested to circle a 0 (not true), 1 (somewhat or sometimes true), and 2 (very true or often true). From the problem items of the YSR, the following eight syndromes were used in this study: Anxious/Depressed, Withdrawn/Depressed, Somatic Complaints, Social Problems, Thought Problems, Attention Problems, Rule-Breaking Behavior, and Aggressive Behavior, and the broad-band scales Internalizing Problems, Externalizing Problems, and Total Problems which are comparable to the same syndromes on the CBCL.

The YSR has been found reliable to use as indicated by the international generalizability of its structure of youth emotional and behavioral problems and has good psychometric properties [24, 26] (see http://www.aseba.org). It has also been found to be suitable for use in the Kenyan population of youths [18]. The Swahili version of the YSR was obtained from the author of the ASEBA. Psychometric findings for the sample in this study are reported below.

#### Procedures for Data Collection

The research proposal was approved by the scientific and ethical review committee of the Faculty of Behavioral and Movement Sciences of Vrije Universiteit Amsterdam. Further approval was received from the National Commission for Science, Technology and Innovation (NACOSTI) in Kenya, which also approved the ethical standards of the study and issued the needed research permit. Approval for data collection was also obtained from the Kiambu and Nyeri county commissioners, county education officers, and district commissioners. Meetings were then scheduled with the local chiefs, village elders, and potential participants in sampled locations, where they were informed about the study and the reasons for carrying out the research. For participants to be recruited in the study, we first established eligibility of the family based on the following: (a) age (6–18 years) and gender of the child; (b) parental or custody relationship of the adult participant with the child (established by cross-checking the parent’s identification card and the child’s birth certificate); and (c) Swahili language competence of the participant in reading and understanding. This was established by having them read the first question of the survey and asking for a response.

Data collection was performed by four trained and research assistants who were supervised by the researcher. Once the eligibility of the participants was established, participants were given informed consent forms to read and sign if they accepted to take part in the study. Any questions about the study and related to their participation were answered by the researcher. Participants who consented to the study were then given questionnaires to complete. The participants were requested to provide honest responses and their confidentiality was assured. The parents and youths filled in the questionnaires independently and gave them back to the researcher after completion. The participants were encouraged to respond to all the items in the questionnaires and the researcher had an opportunity to answer the respondent’s queries during and after completion of the questionnaire. For those who appeared to have difficulty reading and understanding the written Swahili text, items were read aloud by the research assistant, who also filled out the forms assuming that difficulties in reading also imply difficulties in writing. If needed item content was clarified by simple examples provided in the CBCL/YSR manual. After filling in the questionnaire, the participants were debriefed about the study and given a leaflet with additional information about the study. Both the parents and adolescents were given a solar lamp as a token of appreciation for their participation.

### Statistical Analysis

First, descriptive statistics including means, standard deviations, and percentages were computed to examine the demographic characteristics of the participants.

Secondly, using the instructions of the ASEBA manual [24], raw scores for the three broad-band scales and the eight narrow-band syndrome scales were computed by summing the ratings (0, 1, 2) for the items in each scale. To assess the internal consistency of the CBCL and YSR, Cronbach’s α was computed for each instrument’s scale. To assess the applicability of the scales’ structure in this Kenyan sample, confirmatory factor analyses were performed using the set-ups suggested by Ivanova et al. [27, 28] and Harder et al. [18] (L.A. Rescorla, Personal Communication, June 2017). Separate CFA models were estimated for each scale of the CBCL and YSR using MPlus Version 7 [29]. With categorical indicators, the default estimator WLSMV was used to estimate CFA models. Models were assessed for goodness of fit using CFI, TLI, RMSEA, and WRMR. Comparative fit of consecutive adjusted models was tested using Chi square, if needed.

To assess the effect of demographic variables (age groups 6–11 and 12–18, and gender) on EBP reported by parents and adolescents, analyses of variance (ANOVA) with gender and age group as factors were conducted on each CBCL and YSR scale. To calculate the prevalence rates of emotional and behavioral problems, we used the cut-off scores based on multi-cultural norms as described by Achenbach and Rescorla [30]. For each age group, a borderline cut-off point for the specific syndrome scale was determined as the minimum raw score corresponding with a *T*-score between 65 and 69 (93rd–97th percentiles) and a *T-*score between 60 and 63 (84th–90th percentiles) for internalizing, externalizing, and total problems. The clinical cut-off points corresponding for with *T-*score ≥ 70 (98th percentile) for the specific syndrome scales while for internalizing, externalizing and total problems, the scores correspond to a *T*-score ≥ 64 (92nd percentile).

## Results

Demographic characteristics of participants are presented in Table 1. Of the 1068 parents and 576 adolescents, 46 adolescents (8.0% of target sample) and their parents (4.3% of target sample) could not be reached as either the children were in boarding schools or their parents were away during the time of interview. Of the 1022 children and adolescents on whom information was obtained in this study 50.4% were girls while 49.6% were boys, 47.5% were between 6 and 11 years, while 52.2% were between 12 and 18 years. Ninety-six percent of the adult participants were of Kikuyu ethnicity, 1% were Kamba, 0.1% were Luo, while 0.5% were Luhya. In 98% of the cases, the parent had Christian religious affiliations, 0.1% were Muslims, 0.3% were not affiliated with any religion, while 0.1% had other religious affiliations. Ninety-five percent of the youth participants were not married, while 1.8% were married. Almost all the youth participants (95.9%) were students, 0.5% were self-employed, 0.1% were formally employed, 1.7% were not employed, and 0.3% had other types of occupations. Ninety-five percent of the youth participants were attending a school while 1.5% were not in school. Most of the participants (75%) had primary school education as their current or completed level of education, 22.5% had secondary education as their current or completed level of education, 0.2% were not educated, 0.5% had vocational training as their current or completed level of education, 0.3% had college education, and 0.4% were currently pursuing their university education.

**Table 1:** Demographic characteristics of participants

Demographic characteristics	Children	Parents
	N (%)	N (%)
Total *N*	1022	1022
Gender		
Male	507 (49.6)	0 0
Female	515 (50.4)	1022 (100)
Age		
6–11	489 (47.8)	0 (0)
12–18	533 (52.2)	0 (0)
20–40	0 (0)	610 (59.7)
41–60	0 (0)	259 (25.3)
61–80	0 (0)	27 (2.6)
81 and above	0 (0)	1 (0.1)
Ethnic group^a^		
Kikuyu		981 (96.0)
Kamba		10 (1.0)
Luo		1 (0.1)
Luhya		5 (0.5)
Other		16 (1.6)
Religious affiliations^a^		
Christian		1002 (98.0)
Muslim		1 (0.1)
None		3 (0.3)
Other		1 (0.1)
Civic status		
Married	18 (1.8)	726 (71.0)
Not married	979 (95.8)	220 (21.5)
No response	25 (2.4)	76 (7.4)
Occupation		
Student	980 (95.9)	0 (0)
Housewife	0 (0)	515 (50.4)
Self-employed	5 (0.5)	283 (27.7)
Employed	1 (0.1)	91 (8.9)
None	17 (1.7)	28 (2.7)
Other	3 (0.3)	88 (8.6)
Current schooling		
Yes	975 (95.4)	NA
No	15 (1.5)	NA
No response	32 (3.1)	NA
Number of children in houshold^a^		
1–2		351 (35.4)
3–4		465 (46.9)
5–6		170 (17.1)
7–8		4 (0.4)
9–10		1 (0.1)
Other		1 (0.1)
Relationship with child		
Mother	NA	902 (88.3)
Step-mother	NA	8 (0.8)
Grandmother	NA	61 (6.0)
Aunt	NA	15 (1.5)
Guardian	NA	17 (1.7)
Other	NA	1 (0.1)
Level of education		
None	2 (0.2)	34 (3.4)
Primary	766 (75.0)	572 (56.5)
Secondary	230 (22.5)	330 (32.6)
Vocational training	5 (0.5)	19 (1.9)
College	3 (0.3)	32 (3.2)
University	4 (0.4)	14 (1.4)
Other	1 (0.1)	0.0 (00)
No response	11 (1.1)	19 (1.9)

^a^Same for both respondents

All the 1022 adult participants were female with a mean age of 38.2 (SD = 9.5). Seventy-one percent of the adult participants were married while 21.5% were single caretakers. Most of the adult participants (88.3%) were mothers, 0.8% were step-mothers, 6% were grandmothers, 1.5% were aunts, while 1.7% were guardians of the child. The modal number of children per household was three to four. Half of the participants (50.4%) were housewives, 27.7% were self-employed, 8.9% were formally employed, while the rest did not give information about their employment status. Fifty-six percent of the adult participants had completed primary school education, 32.6% had secondary school education, 1.9% had vocational training, 3.2% had college training, 1.4% had university education, while 3.4% had no formal education.

### Fit Indices of the Eight-Syndrome CBCL and YSR Structure

Table 2 (upper panel) shows model fit indices for the CBCL scales obtained from CFA. First, models were assessed for goodness of fit to the whole sample, and the analyses were repeated for the two age groups (6–11, and 12–18 years) using the MPlus multi-group function. Three of the CBCL scales (Withdrawn/Depressed, Social Problems, and Attention Problems) appeared to have an excellent fit to their default models, while good to excellent fit was found for the other scales by allowing one (for Rule-Breaking and Aggressive Behavior) or two (for Anxious/Depressed, Somatic Complaints, and Thought Problems) correlated residuals in the model.

**Table 2:** Model fit indices obtained from CFA of CBCL and YSR scales

	χ^2^	df	CFI	TLI	RMSEA	95% CI		p	WRMR
						LL	UL		
CBCL scales									
Anxious/depressed	197.844	63	0.944	0.931	0.046	0.039	0.053	0.826	1.216
Withdrawn/depressed	54.548	20	0.952	0.933	0.041	0.028	0.054	0.858	0.943
Somatic complaints	121.600	42	0.975	0.968	0.043	0.034	0.052	0.894	1.049
Social problems	48.849	27	0.975	0.967	0.028	0.015	0.041	0.999	0.0816
Thought problems	183.962	88	0.961	0.954	0.033	0.026	0.039	0.999	1.028
Attention problems	103.221	35	0.964	0.954	0.044	0.034	0.054	0.849	1.066
Rule-breaking behavior	239.458	118	0.943	0.934	0.032	0.026	0.038	0.999	1.072
Aggressive behavior	392.484	134	0.973	0.969	0.043	0.038	0.048	0.985	1.317
YSR scales									
Anxious/depressed	100.587	54	0.956	0.947	0.040	0.028	0.052	0.905	0.927
Withdrawn/depressed	32.093	14	0.929	0.894	0.049	0.027	0.072	0.486	0.817
Somatic complaints	65.700	35	0.973	0.965	0.041	0.025	0.056	0.841	0.854
Social problems	83.810	44	0.938	0.923	0.041	0.028	0.055	0.855	0.903
Thought problems	79.435	54	0.955	0.945	0.030	0.014	0.043	0.995	0.844
Attention problems	53.856	27	0.948	0.930	0.043	0.026	0.060	0.729	0.859
Rule-breaking behavior	95.568	65	0.973	0.968	0.030	0.016	0.042	0.998	0.822
Aggressive behavior	164.791	103	0.952	0.944	0.034	0.024	0.043	0.999	0.955

Fit indices for multi-group data (CBCL) and item loadings (CBCL and YSR) can be obtained from the third author

*χ*^*2*^ chi square, *df* degrees of freedom, *CFI* comparative fit index, *TLI* Tucker-Lewis Index, *RMSEA* root mean square error of approximation, *95% CI* 95% confidence interval of RMSEA, *LL* lower limit of RMSEA, *UL* upper limit of RMSEA, *p* p value, *WRMR* weighted root mean square residual

Table 2 (lower panel) shows model fit indices for the YSR scales obtained from CFA. Separate CFA models were estimated for each scale, and four scales (Somatic Complaints, Social Problems, Thought Problems, and Attention Problems) appeared to have a very good fit to their default models, while good to excellent fit was found for Withdrawn/Depressed and Aggressive Behavior by allowing one correlated residual in the model. However, best fit was reached for Anxious/Depressed only by deleting item 32 (*I feel that I have to be perfect*), and for Rule-Breaking Behavior only by deleting items 72 (*I set fire*) and 99 (*I smoke, chew or sniff tobacco*).

### Internal Consistency of CBCL and YSR Reports

The internal consistency of CBCL and YSR problem scales was examined using Cronbach’s alpha. As shown in Table 3, excellent Cronbach’s alphas were found for the CBCLs three broad-band scales internalizing (0.85 and 0.83), externalizing (0.86 and 0.80), and total problems (0.95 and 0.93), for ages groups 6–12 and 12–18, respectively. Relatively low alphas were found for the syndrome scales withdrawn/depressed (0.58 and 0.67), social problems (0.62 and 0.57), and rule-breaking (0.67 and 0.57), and acceptable alphas were found for anxious/depressed (0.71 and 0.67), somatic complaints (0.78 and 0.78), thought problems (0.74 and 0.65), attention problems (0.73 and 0.70), and aggressive behavior (0.81 and 0.76).

**Table 3:** Cronbach’s alpha internal consistency of CBCL and YSR problem scales

Scales	CBCL 6–11	CBCL 12–18	YSR 12–18
	(N = 489)	(N = 533)	(N = 533)
Anxious/depressed	0.71	0.67	0.63
Withdrawn/depressed	0.58	0.51	0.50
Somatic complaints	0.78	0.78	0.73
Social problems	0.62	0.57	0.64
Thought problems	0.74	0.65	0.59
Attention problems	0.73	0.70	0.63
Rule-breaking	0.67	0.57	0.49
Aggressive behavior	0.81	0.76	0.65
Internalizing	0.85	0.83	0.81
Externalizing	0.86	0.80	0.70
Total problems	0.95	0.93	0.89

Most Cronbach’s alphas for the YSR were comparable to the CBCL alphas in this study. Those for the three broad-band scales were acceptable to excellent; internalizing (0.81), externalizing (0.70), and total problems (0.89). Relatively low alphas were found for the syndrome scales withdrawn/depressed (0.50), social problems (0.64), rule-breaking (0.49), anxious/depressed (0.63), thought problems (0.59), attention problems (0.63), and aggressive behavior (0.65). Acceptable alphas were found for somatic complaints (0.73).

### CBCL and YSR Problem Behavior Scores

Mean CBCL scores and standard deviations for problem behaviors by gender and age group are presented in Table 4. ANOVA was conducted to examine the influence or impact of gender (boys, girls) and age group (ages 6–11 and 12–18 years) on parent-reported emotional and behavioral problems. Younger children had significantly higher scores on all problem scales except for anxious/depressed, withdrawn/depressed, somatic complaints, and internalizing problems. However, no significant gender or gender by age group differences were found.

**Table 4:** Mean (SD) CBCL scale scores by gender and age

	All	Boys		Girls		Gender		Age group		Gender × age	
		6–11	12–18	6–11	12–18	F	p	F	p	F	p
N	1022	243	263	245	269						
Anxious/depressed	3.5 (3.7)	3.7 (3.6)	3.2 (3.1)	3.6 (3.4)	3.6 (3.4)	0.81	0.37	1.55	0.21	1.63	0.20
Withdrawn/depressed	2.3 (2.4)	2.3 (2.6)	2.0 (2.2)	2.3 (2.5)	2.4 (2.2)	1.25	0.26	0.86	0.35	1.25	0.26
Somatic complaints	2.4 (3.2)	2.3 (3.2)	2.2 (3.0)	2.7 (3.3)	2.6 (3.2)	3.21	0.07	0.02	0.89	0.64	0.42
Social problems	3.5 (3.2)	3.7 (3.5)	3.0 (2.8)	3.9 (3.3)	3.4 (3.1)	2.22	0.14	8.09	0.005	1.17	0.73
Thought problems	2.6 (3.4)	3.0 (3.9)	2.4 (3.0)	2.9 (3.8)	2.2 (2.8)	0.48	0.49	10.45	0.001	0.10	0.76
Attention problems	3.0 (3.2)	3.6 (3.8)	2.6 (2.9)	3.0 (3.1)	2.7 (3.1)	1.10	0.29	11.53	0.001	2.26	0.12
Rule-breaking	2.6 (3.1)	3.1 (3.6)	2.3 (2.8)	2.7 (2.4)	2.6 (2.7)	0.13	0.71	5.01	0.030	2.63	1.10
Aggressive behavior	3.8 (4.7)	4.3 (5.4)	3.0 (3.8)	4.2 (5.0)	3.6 (4.4)	0.50	0.48	10.95	0.001	1.30	0.26
Internalizing	8.2 (7.6)	8.3 (8.5)	7.3 (6.8)	8.5 (7.6)	8.7 (7.3)	2.29	0.13	0.65	0.420	1.61	0.20
Externalizing	6.4 (7.2)	7.4 (8.5)	5.3 (5.9)	6.9 (7.8)	6.1 (6.6)	0.09	0.77	9.68	0.002	2.08	0.15
Total problems	28.6 (25.2)	31.5 (29.6)	25.0 (21.6)	30.8 (26.1)	27.3 (22.8)	0.22	0.64	9.86	0.002	1.03	0.31

F-values are based on Anova by gender and age

Mean YSR scores and standard deviations for problem behaviors by gender are presented in Table 5. ANOVA was conducted to investigate the effect of gender on problem scores. Girls appeared to score significantly higher on the narrow-band scales somatic complaints and attention problems, on both broad-band scales internalizing and externalizing, and on total problems.

**Table 5:** Mean (SD) YSR scale scores by gender

Scales	All	Boys	Girls	F	p
		n = 263	n = 269		
Anxious/depressed	4.9 (3.3)	4.7 (3.0)	5.1 (3.6)	1.58	0.21
Withdrawn/depressed	3.4 (2.4)	3.3 (2.2)	3.5 (2.5)	0.61	0.44
Somatic complaints	2.86 (3.0)	2.20 (2.6)	3.5 (3.3)	25.6	0.00
Social problems	3.1 (3.0)	2.9 (2.7)	3.2 (3.2)	1.76	0.19
Thought problems	3.1 (2.8)	2.9 (2.6)	3.2 (3.1)	1.04	0.31
Attention problems	1.86 (2.4)	1.62 (2.15)	2.1 (2.6)	5.11	0.02
Rule-breaking	2.5 (2.9)	2.3 (2.6)	3.3 (3.2)	3.11	0.08
Aggressive behavior	3.5 (3.5)	3.2 (3.2)	3.8 (3.7)	3.16	0.08
Internalizing problems	11.2 (7.3)	10.2 (6.6)	12.0 (8.0)	8.20	0.00
Externalizing problems	6.0 (5.8)	5.5 (5.3)	6.5 (6.2)	3.78	0.05
Total problems	35.50 (20.94)	33.12 (18.10)	37.83 (23.19)	6.82	0.01

The cross-informant agreement in this study was low with a mean intra-class correlation coefficient (ICC) of 0.146 (range − 0.013 to 0.239) across the CBCL and YSR narrow-band and broad-band scales, and a mean cross-informant Pearson correlation of 0.09 (range 0.02–0.15).

### Prevalence of Emotional and Behavioral Problems in Kenyan Children and Adolescents

Based on the mean total problem scores obtained in this study for both CBCL and YSR, problem levels in this Kenyan sample were compared to multicultural norms and found to compare to group 2 (middle scoring group norm) [30]. To obtain the prevalence rates of elevated scores for each scale based on parent and adolescent reports, the multicultural group norm 2 cut-off scores for borderline and clinical scores were used to define children and adolescents scoring in the borderline or clinical range, respectively [30]. Frequencies and percentages were then used to examine the prevalence rates for each syndrome scale. Table 6 shows the proportion of children and adolescents with syndrome scale scores in the borderline and clinical range.

**Table 6:** Prevalence of emotional and behavioral problems in the borderline and clinical range based on multi-cultural norms (cf. [30])

		Above borderline cutoff point		Above clinical cutoff point	
		n	%	n	%
Anxious/depressed	C	129	12.6	52	5.1
	Y	41	4.0	16	1.6
Withdrawn/depressed	C	147	14.4	50	4.9
	Y	45	4.4	15	1.5
Somatic complaints	C	199	19.5	121	11.8
	Y	47	4.6	17	1.7
Social problems	C	170	16.6	72	7.0
	Y	46	4.5	27	2.6
Thought problems	C	175	17.1	107	10.5
	Y	22	2.2	10	1.0
Attention problems	C	59	5.8	24	2.3
	Y	5	0.5	2	0.2
Rule-breaking	C	110	10.8	62	6.1
	Y	21	2.1	5	0.5
Aggressive behavior	C	76	7.4	26	2.5
	Y	13	1.3	2	0.2
Internalizing problems	C	307	30.0	197	19.3
	Y	122	11.9	54	5.3
Externalizing problems	C	164	16.0	104	10.2
	Y	34	3.4	20	2.0
Total problems	C	276	27.0	172	16.8
	Y	77	7.5	42	4.1

*C* based on CBCL problem scores, N = 1022, *Y* based on YSR problem scores, N = 533

Based on the multicultural norms used (93rd and 84th percentiles for the narrow- and broad-band scales, respectively), the Kenyan children and adolescents had high prevalence rates in the borderline clinical range. Specifically, according to parents’ reports, compared to the multicultural norms these children and adolescents scored (at least) two times more often in this range on anxious/depressed, withdrawn/depressed, somatic complaints, social problems, thought problems, internalizing problems, and total problems. On attention problems, rule-breaking and aggressive behavior, and externalizing problems, the percentages with borderline scores were similar to those of the multicultural norms.

Based on the multicultural norms used for the clinical range (98th and 92nd percentile for the narrow and broadband syndrome scales, respectively), Kenyan children and adolescents had high prevalence rates of emotional and behavioral problems. Compared to these norms, parents reported a prevalence rate that is 6 times higher for somatic complaints, 5 times higher for thought problems, 3 times higher for rule breaking, and 2 times higher for anxious/depressed, withdrawn/depressed, internalizing, and total problems. On attention problems, aggressive behavior and externalizing problems, the scores were similar to those of the multicultural norms.

In contrast, based on the multicultural norms developed for the YSR, adolescents in this Kenyan sample had lower prevalence rates on all narrow-band and broad scales for both the borderline and clinical ranges. Most notably, prevalence rates of attention problems, rule-breaking behavior, aggressive behavior, and externalizing problems were well below the expectable levels.

## Discussion

Knowledge of prevalence rates of EBP in children and adolescents provides valuable information that helps in determining what the mental health burden is for children and adolescents in a particular population. Starting from the observation that hardly any reliable information on this topic is available for general population samples of youths living in sub-Saharan countries, the purpose of the Kenya Child and Adolescent Mental Health Study reported here was to establish the prevalence rates of EBP in Kenyan children and adolescents. To address this aim, using a population based age and gender stratified random sample of Kenyan parent and adolescent informants, we first examined the applicability of the scale structure of the Swahili version of the CBCL and YSR scale structure to this population. Then, using these instruments the distribution of EBP across age and gender was described and the prevalence of deviant levels of EBP when compared to multicultural norms.

### Psychometric Properties of CBCL and YSR

The findings from this study suggest that the Swahili translations of the standardized questionnaires CBCL and YSR can be reliably used with Kenyan parent and adolescent informants, respectively, to obtain meaningful information on child and adolescent mental health. The default scale structure [24] of the Swahili versions of both the CBCL and YSR showed a remarkably good fit to the data. However, the CFA of the YSR scales showed that three items did not fit their respective scales Anxious/Depressed and Rule-Breaking Behavior and had to be deleted from the model to reach good fit. Post-hoc analyses showed that indeed item-total correlations of the items *I feel that I have to be perfect*, and *I set fire* and *I smoke, chew or sniff tobacco* were very low (.057, .048, and .077 in their respective scales) suggesting that they are not indicating an emotional or behavioral problem. Indeed, in Kenya children and adolescents are generally required to act or think in a certain way. Failure to stick to the socially allowed norm may lead to being frowned on and seen as disobedience. The question “I set fire” is likely not appropriate in the context of mental health issues as in answering the item most of the youth thought it included burning garbage, or lighting a fire for cooking. Finally, it is very rare to see children and youth between 6 and 18 smoking, chewing, or sniffing tobacco. The few that do may not admit it as selling cigarettes to children under 18 years is illegal in Kenya.

Kenyan parental and youth reports on EBP indicate good to excellent Cronbach’s alphas for both the CBCL’s and YSR’s three broad-band scales (internalizing, externalizing, and total problems). However, relatively low to acceptable alphas were found for the eight narrow—band syndrome scales for both instruments, which is similar to observations on these instruments’ internal consistencies in other countries and cultures. Notably, low Cronbach’s alphas were found for the scales withdrawn/depressed, social problems, and rule-breaking in both instruments (alpha range 0.57–0.67 and 0.49–0.64 for CBCL and YSR respectively). This indicates that the CBCL and YSR items work in a similar manner in Kenya as they work in other parts of the world. Internal consistency reliabilities can be affected by various factors like number of items in a subscale, or homogeneity in response. While researchers often write items to reflect predefined (psychological) constructs that result in homogeneous scales, often at the cost of predictive validity [31]. The items in the CBCL and YSR are based on mental health complaints recorded at intake. This has resulted in relatively heterogeneous scales with moderate internal consistencies, but with a quite replicable structure, also in this study, and good test–retest reliabilities and validity [23, 32].

### Prevalence of Emotional and Behavioral Problems

#### Parent Reports

Younger children had significantly higher scores on all problem scales except for anxious/depressed, withdrawn/depressed, somatic complaints, and internalizing problems. However, no significant gender or gender by age group differences were found. Higher levels of externalizing problems, social problems, and attention problems for younger than for older children have been repeatedly reported. For example, findings from a longitudinal study from age 4 to age 18 [33] demonstrated that the overall levels of problems and externalizing problems decrease with age. Most children learn to outgrow these problems with age [34].

The most noticeable result to emerge from the parent reports is the high prevalence rates of EBP (27 and 16% for borderline and clinical ranges, respectively) in Kenyan children and adolescents when compared to appropriate multicultural norms [30]. These prevalence rates indicate an increase of mental health problems by about a factor 1.6 in Kenyan children and adolescents when compared to their age-mates from other countries. Specifically, according to parents’ reports, compared to the multicultural norms these children and adolescents scored (at least) two times more often in this range on anxious/depressed, withdrawn/depressed, somatic complaints, social problems, thought problems, internalizing problems, and total problems.

Another interesting finding pertains to the high prevalence of rates of deviant scores for internalizing problems when compared to externalizing problems (30 vs. 16% for borderline range and 19.3 vs. 10% for clinical ranges). While these percentages are in the expectable range for externalizing problems, they are almost doubled for internalizing problems. Additionally, higher than expected prevalence rates were reported for somatic complaints and thought problems in this population. These findings support the findings of Weisz et al. [20] who also reported higher under-controlled than over-controlled problems in Kenyan children and adolescents. High internalizing problems may have been partly precipitated by the elevated levels of somatic complaints. The presence of high levels of somatic complaints could be a consequence of Kenyan parents imposing strict obedience rules to their children. Somatic complaints may emerge as a response to distress in their children from this exposure to strict obedience requirements. Thus, the strong tendency towards somatic complaints may be due to psychological stress in Kenyan youth. However, it could also be that parents understand and attend to their children more when they present physical symptoms rather than emotional symptoms. Expression of physical problems may, therefore, free them from the expected duties and associated strictness more than when they present emotional problems.

A speculation to these high prevalence rates could be that some of the children and adolescents in Kenya have been exposed to uncommonly high levels of risk factors associated with emotional and behavioral problems. Some of these risk factors may include infectious or chronic disease [35] and malnutrition [36]; parental psychopathological [37, 38]; poor parent–child connectedness [39]; poor parental monitoring and control [40, 41]; and poverty [42]. Children and adolescents exposed to chronic, high levels of risk factors may be at a high risk of developing emotional and behavioral problems that tend to persist when risk is not taken away.

#### Youth Reports

Girls reported higher levels of somatic complaints and attention problems, and on both broad-band scales internalizing and externalizing, and total problems. The tendency of gender differences in internalizing problems is consistent with another study conducted in Kenya [18] that also found girls to report more internalizing problems than boys, and with the findings from societies across multiple cultures [43]. Among the plausible explanations for these results could be the different gender roles and expectations between boys and girls. For example, in most Kenyan communities, boys are expected to be strong, courageous and independent, whereas girls are expected to be calm, quiet, and exhibit nurturing behaviors. The gender socialization could foster the exhibition of more internalizing problems in girls than in boys. However, by contrast, findings from the youth reports indicated relatively low prevalence rates of elevated levels of EBP when compared to cross-cultural norms (7.5 and 4.1% for borderline and clinical ranges, respectively). Specifically, elevated levels externalizing problems had a low prevalence when based on youth self-reports. Lower prevalence rates may be due to different definitions of behaviors (e.g., quarreling, fighting), or to reluctance to reports certain behaviors. Similar results were found when comparing teacher- and youth self-reports for Moroccan immigrant youth in the Netherlands to those for Dutch or Turkish immigrant youth [44].

#### Parent Reports Versus Youth Reports

In this study, low cross-informant agreement was found compared to the cross-informant agreement reported by other studies. The average Kenyan parent and youth cross-informant *r*s were smaller than the mean *r* of 0.25 reported in the review study by Achenbach et al. [45] and the mean *r* of 0.48 (range 0.27–0.56) reported in a Dutch study [46]. Generally, researchers agree that parents tend to report fewer problems compared to their children’s self-reports, which was also confirmed in this study. However, the low parent-adolescent agreement in this sample could be because these informants have different perspectives of the child’s levels of functioning that may be appropriate each in their own right. For example, lack of agreement of reports of internalizing problems could be because the parents may not be aware of their children’s emotional problems [38]. The lack of this knowledge may be a result of adolescents confiding less in their parents; poor communication patterns between parents and their children; or perhaps in Kenyan communities, parents rarely discuss with their children about their emotional problems. This was further demonstrated during the data collection process when some parents stated that “…the interview gave me an opportunity to think about my child…I’ve never thought about my child in this way”. Another reason may be the different views of parents on their adolescents’ behavior. Weisz et al. [20] reported that Kenyan parents value obedience and politeness. This could have made the difference in reporting about emotional problems. Since parents regard inhibited behavior (being quiet, avoiding arguments, etc.) as desirable, they may not view strongly inhibited behaviors as potentially indicating an emotional problem and hence are less likely to report them.

### Strengths and Limitations of the Study

This study has several strengths. First, to our knowledge, this is the first general population based study that has been conducted in Kenya to investigate the prevalence of EBP using standardized measures in a large sample of children and adolescents from the general population. Therefore, the current study does not only increase our scientific knowledge about the prevalence of emotional and behavioral problems in Kenyan youth, but also provides normative data for emotional and behavioral problems in this population. Secondly, the study is based on sound methodology with random selection of study locations, a large sample size with a well-balanced distribution across age and gender, and the use of well-validated instruments (CBCL, YSR), making the findings of this study possibly generalizable not only to Kenya but also to other African countries with similar cultural background as Kenya. Thirdly, our study takes into account both the adolescent child and their caregiver’s reports. The study, therefore, captures not only the adolescents’ feelings and behavior but also their parents’ reports of their behavior, which is important as parents are usually those responsible for seeking help for child mental health problems. Additionally, the study taps on both emotional and behavioral problems. This is a major step in identification of children who may need professional help and facilitating the implementation of important policy decisions to improve the well-being of these children. Lastly, the comparison of EBP in Kenyan children with multicultural norms based on the standardized instruments gives us insight about the state of mental health of Kenyan children and adolescents compared with their counterparts in other countries.

However, the study findings should be interpreted in light of several limitations. To begin with, the study was only conducted in two out of the 42 counties in Kenya. Secondly, the instruments used in this study were not yet validated against certified mental health diagnoses or other data about the child’s functioning. Additionally, the reports of other informants (e.g., fathers and teachers) were not taken into account. In general, fathers may provide valuable information about their children provided they are substantially involved in their children’s lives. Similarly, teachers may give important insight into the child’s emotional and behavioral problems without biases that influence parent and adolescent reports. However, although teachers may be well-aware of children having very severe problems, the accuracy of teachers’ reports in general in the typical Kenyan situation may be doubted given that Kenyan teachers have to care about huge classes of 40–60 children. Therefore, they may not be so much aware of every child’s behaviors and feelings.

### Study Implications and Conclusion

Our study findings have important implications for signaling, detection, counseling, referral, treatment, and public mental health policy regarding emotional and behavioral problems in Kenyan children and adolescents. As recently noted by Ndetei et al. [47], the Kenya Mental Health Bill 2014 has been drafted and is in parliament awaiting enactment into law, hopefully bringing Kenya’s mental health services to a par with international standards. The country has also adopted the World Health Organization’s Global Mental Health Action Plan 2013–2020, who’s objectives are to ensure effective leadership and governance of mental health services, to provide mental and social care services in community-based settings, to implement strategies for the promotion of mental health and the prevention of mental ill-health, and to strengthen information systems and research in mental health. However, although in the proposed bill children and youth are mentioned as targets of mental health interventions in primary care, the country’s awareness of child/adolescent mental health problems seems still limited to mainly intellectual disability, autism and ADHD. Although in several secondary schools teachers have been trained to counsel students with school-related problems, they hardly have options for referral for mental health services provided by professionals. Mental health illiteracy also seems to hamper detection and treatment of EBP by gatekeepers like parents, general health professionals and paraprofessionals and other adults who regularly are confronted with youth in trouble.

Given this situation, the results of this study are very relevant as they suggest that EBP are highly prevalent in the population of Kenyan children and adolescents. Early identification, screening, and treatment of children with emotional and behavioral problems is a task that should be given priority in Kenyan children to avoid the risk of developing future functional and mental health problems. The instruments used in this study may prove helpful in this early identification and screening, as well as for the assessment of intervention outcomes. Particular attention is needed in regards to the high prevalence of somatic complaints, and their potential meaning as signals of emotional problems. If this is confirmed it is important that, based on these signals, mental health providers in Kenya apply appropriate prevention and intervention measures necessary to mitigate these problems. To raise public awareness and enhance early signaling, detection and appropriate referral for services, information and training may be offered to all those who are responsible for the well-being of children and adolescents. These may include parents, teachers, family physicians, and professionals and para-professionals working in general health facilities and in justice systems. Additionally, policy makers should take these findings into consideration and implement policies that promote access to mental health care services to children and adolescents with EBP. Moreover, future empirical studies focusing on EBP should investigate the risk factors related to EBP. This will provide useful insights into malleable risk factors for emotional and behavioral problems in Kenyan children and youths, which form an essential basis for prevention of and reducing the prevalence of behavioral and emotional problems in this population. It is also necessary to explore the need for help assessment in these communities. Since additional risk factors data and need for support assessment is also available in the Kenya Child and Adolescent Mental Health Study, our next task will be to examine these important aspects.

In summary, this study explored the prevalence of emotional and behavioral problems in Kenyan children and adolescents. The study provides strong evidence of elevated levels of parent-reported EBP. Mental health providers should focus on interventions that reduce EBP in Kenyan children and adolescents to avoid the risk of future mental health problems and malfunction. Additional studies are required to examine the risk factors to EBP in Kenyan children and adolescents and assess the need of help for these problems.

## Summary

Emotional and behavioral problems (EBP) during childhood and adolescence are a common concern for parents and mental health stakeholders as they predict numerous problematic outcomes in adulthood. However, little has been documented about the prevalence of EBP in sub-Saharan African countries, including Kenya. This study aimed to establish the prevalence rates of EBP in children and adolescents in Kenya using standardized questionnaires. The study included reports from 1022 Kenyan parents and 533 adolescents included through multistage random sampling from the country’s Central Province. Parents with children between 6 and 18 years completed the Child Behavior Checklist (CBCL) [23], while the adolescents (ages 12–18) completed the Youth Self-Report (YSR) [26]. Results from confirmatory factor analyses suggest that the Swahili translations of the standardized questionnaires CBCL and YSR can be reliably used with Kenyan parent and adolescent informants, respectively, to obtain meaningful information on child and adolescent mental health. The default scale structure [24] of the Swahili versions of both the CBCL and YSR showed a remarkably good fit to the data. Results showed further that EBP are 1.6 times more prevalent in Kenyan children and adolescents than in others compared to multi-cultural standards [30]. Based on parent reports 27 and 17% were scored in the borderline and clinical range, respectively. Younger children were scored higher on EBP than older children, and higher on internalizing problems in particular. Expectable elevated levels of externalizing problems in boys were not found. Based on youth self-reports girls scored higher than boys, especially on internalizing problems. The study provides evidence on elevated parent-reported EBP in Kenyan youths, specifically elevated internalizing problems. This information could be used to set up mental health provisions and interventions for Kenyan youths.
